# Effect of Surface Pt Doping on the Reactivity of Au(111) Surfaces towards Methanol Dehydrogenation: A First-Principles Density Functional Theory Investigation

**DOI:** 10.3390/molecules28237928

**Published:** 2023-12-04

**Authors:** Merve Demirtas, Hande Ustunel, Daniele Toffoli

**Affiliations:** 1 Department of Physics, Middle East Technical University, Dumlupinar Blvd. 1, 06800 Ankara, Turkey; merve.demirtas@tubitak.gov.tr; 2 Network Technologies Department, Tubitak Ulakbim, 06800 Ankara, Turkey; 3 Dipartimento di Scienze Chimiche e Farmaceutiche, Università degli Studi di Trieste, Via L. Giorgieri 1, I-34127 Trieste, Italy; 4 IOM-CNR, Istituto Officina dei Materiali-Consiglio Nazionale delle Ricerche, S.S. 14, km 163.5, I-34149 Trieste, Italy

**Keywords:** density functional theory, methanol dehydrogenation reaction, nudged elastic band, activation energy, reaction energy

## Abstract

The surprisingly high catalytic activity of gold has been known to the heterogeneous catalysis community since the mid-1980s. Significant efforts have been directed towards improving the reactivity of these surfaces towards important industrial reactions. One such strategy is the introduction of small amounts of other metals to create Au-based surface alloys. In this work, we investigated the synergistic effect of the Pt doping of a Au(111) surface on decreasing the activation barrier of the methanol dehydrogenation elementary step within first-principles density functional theory. To this end, we constructed several models of Pt-doped Au(111) surfaces, including a full Pt overlayer and monolayer. The effect of Pt surface doping was then investigated via the computation of the adsorption energies of the various chemical species involved in the catalytic step and the estimation of the activation barriers of methanol dehydrogenation. Both the electronic and strain effects induced by Pt surface doping substantially lowered the activation energy barrier of this important elementary reaction step. Moreover, in the presence of preadsorbed atomic oxygen, Pt surface doping could be used to reduce the activation energy for methanol dehydrogenation to as low as 0.1 eV.

## 1. Introduction

The selective oxidation of alcohols is routinely used in the industrial synthesis of a large number of important chemicals, such as aldehydes, esthers, and ketones [1], whose production can be selectively favored throughout the reaction steps leading to full oxidation [2]. In addition, the oxidation of alcohols via electrochemical reactions is used in fuel cells [3]. A large number of metals including Pt [4], Pd [5], and Ag [6] and their alloys [7,8] have been used successfully as catalysts in oxidation reactions. However, the most unexpected discovery in this field has been the catalytic properties of nanosized Au, whose reactivity in the form of ultrafine particles was proven in 1987 by Haruta et al. [9]. Since then, the catalytic properties of Au in both bulk [3,7] and particle form [10,11] have been continuously investigated.

While making use of their low environmental cost [12], resistance to poisoning [13], and selectivity [14], the activity of Au catalysts can be improved by several methods, including the introduction of surface atomic oxygen [14] and the tuning of the particle size [15,16]. Another method based on material design is to utilize the ensemble effects that emerge through alloying the surface with other metals. There is evidence that both alloys [17] and mixtures [18] of Au with other metals outperform pure Au. Of particular interest is the work by Zhao et al. [18], who discovered via measurements of the conversion of 5-hydroxymethylfurfural that while AuPd alloys perform better than Au, a simple Au/Pd mixture gives an even better conversion rate than the alloy. In a detailed density functional theory (DFT) study by Li and Henkelman [19], the reactivity of a Au(111) surface decorated with Pd and Rh patterns of different shapes and sizes was investigated, and a clear inverse relationship between the Pd or Rh surface concentration and ethanol dehydrogenation reaction barrier was discovered. Yi et al. synthesized NiAu nanoparticles of different sizes and structures and observed a five-fold increase in benzylalcohol oxidation compared to supported Au particles [15].

Alloys of Au with many metals including Pd [7,18,20], Rh [19], Ag [21], Ni [15], Ru [22], and Ir [22] have been investigated experimentally and theoretically regarding their potential in the selective oxidation of alcohols. Conversely, although Pt is known to catalyze C-H bond breaking, which tends to be a rate-determining step for the partial oxidation of alcohols [23], Au-Pt alloys have received limited attention in this context. However, a noteworthy experimental study by Tenney et al. [24] revealed that the activity of bimetallic AuPt clusters (with a Au content greater than 50%) was similar to that of pure Pt. In another more recent experimental work, Stepanova et al. [25] observed that the introduction of Au into supported Pt clusters led to an increase in the propane conversion without affecting the selectivity towards propylene. The atomistic origins of these interesting synergistic outcomes have yet to be uncovered.

In this paper, our objective was to explore, within the planewave pseudopotential DFT framework and at the atomistic scale, the catalytic activity of Pt atoms in the form of substitutional surface alloys with Au. Our model surface calculations aimed to explore the methanol dehydrogenation reaction in both the presence and absence of surface atomic oxygen species. While atomic oxygen is known to drastically reduce the activation barrier for dehydrogenation reactions, the pretreatment of the metal surface may prove difficult [26]. We performed this investigation by considering the activation energy for the following reactions:

(1) $$CH_{3}OH(ads)\to CH_{3}O(ads)+H(ads)$$

and

(2) $$CH_{3}OH(ads)+O(ads)\to CH_{3}O(ads)+OH(ads),$$

where the designation (ads) indicates that the species are all adsorbed on the surface.

Several different reaction pathways for methanol dehydrogenation on extended metallic surfaces and nanostructures are possible, depending on whether the initial bond activation involves an O–H bond (the alkoxy path) or a C–H bond (the hydroxyalkyl path). The question of whether H abstraction from OH- or CH_3_- groups represents the bottleneck at the beginning of the oxidation reactions must be addressed separately for each catalyst used [7,27]. In fact, depending on the catalyst, as demonstrated in the work of Wu and Wang [28], C–H bond breaking may have a larger activation barrier than O–H bond breaking. In this work, we only focused on O–H bond scission and compared different Pt-enhanced Au catalysts for this specific step of the reaction mechanism. Theoretical studies of the O-assisted selective coupling of CH_3_OH on gold surfaces [29] and several DFT works on (100) and (310) Au surfaces [7,30] have considered the alkoxy path; we concentrated on this elementary reaction step in this work and used it as a probe reaction to understand the effect of Pt surface doping on Au(111) surfaces.

In addition to a bare Au(111) surface, serving as the benchmark, we considered surface alloys with Pt:Au ratios of 1:8 (1 Pt/Au), 1:4 (2 Pt/Au), 1:2 (3 Pt/Au), and 1:1 (corresponding to a surface Pt monolayer), and a full subsurface Pt layer just below the topmost Au layer. These ratios refer not to the bulk but only to the nine surface atoms of our 3 × 3 surface slab. The phase diagrams [31] and segregation tendencies [32] of this alloy in different morphologies suggest that such a surface alloy would indeed be thermodynamically stable. For each Pt concentration, we calculated the adsorption energies of CH_3_OH, CH_3_O, OH, and atomic O and H together with the energetics of CH_3_OH/H, CH_3_OH/O, and CH_3_O/OH coadsorption. Understanding the adsorption and coadsorption energies is important since there may be synergistic stabilizing effects, whereby the total adsorption energy may be greater than the sum of the individual energies [16]. Furthermore, we calculated the activation barrier of the reactions shown in Equations (1) and (2) on all model surfaces. The plan of the paper is the following: In Section 2, we give the computational details. Section 3 discusses the most important results of this study, while the conclusions are reported in Section 4.

## 2. Computational Details

The calculations were performed using the planewave pseudopotential density functional theory [33,34] method within the gradient-corrected approximation (GGA) as implemented in the open-source Quantum Espresso [35] code suite. The Perdew–Wang (PW-91) exchange-correlation [36] functional was used to approximate electron–electron interactions. The open-source program XCrysDen [37] was used for visualization and to produce the figures. During BFGS (Broyden–Fletcher–Goldfarb–Shanno) geometry optimizations, a force threshold per atom of 0.001 eV/Å was used. The use of ultrasoft pseudopotentials [38] to model the interaction between atomic nuclei and electrons allowed an affordable kinetic energy cutoff of 40 Ryd and a density cutoff of 400 Ryd. With these parameters, the fcc Au and Pt lattice constants were calculated to be 4.16 Å and 3.99 Å, in good agreement with the measured values of 4.08 Å [39] and 3.91 [39], respectively.

The Au(111) surface was represented by a slab model using a simulation cell of 3 × 3 unit cells consisting of four layers repeating the ABCABC stacking. The two layers at the bottom were held fixed to mimic bulk behavior, while the other two were allowed to move freely during geometry optimization. In order to reduce the interactions between successive slabs, the vacuum separation was set to be at least 14 Å in all calculations. A Monkhorst–Pack *k*-point mesh of 4×4×1 was used to compute the Brillouin zone integrals [40].

The adsorption energies of atomic or molecular species were calculated using

(3) $$E_{b}=E_{tot}-E_{slab}-E_{ads},\;$$

where $E_{tot}$ is the total energy of the surface slab with the adsorbate; $E_{slab}$ is the total energy of the surface slab; and $E_{ads}$ is the total energy of the isolated adsorbate, which was calculated in a large cubic simulation box at the Γ point. With this definition, negative adsorption energies correspond to stable adsorption configurations. For the coadsorbed species, the adsorption energy was calculated using

(4) $$E_{b}=E_{tot}-E_{slab}-E_{ads,1}-E_{ads,2},\;$$

where the definition of $E_{b}$, $E_{tot}$, and $E_{slab}$ are the same as in Equation (3), while $E_{ads,1}$ and $E_{ads,2}$ are the energies of the two isolated adsorbates, calculated separately. All surfaces were geometry-optimized prior to adsorption.

The transition states and the activation energies were calculated using the climbing image nudged elastic band method (CI-NEB) [41]. The reaction path was divided into 7 images including the endpoints; however, in a few cases this number was increased to 9 to speed up convergence. Once the transition state was identified, no further optimizations were conducted. Finally, partial density of states (PDOS) and Bader partial charge analyses, as implemented by the Henkelman group [42], were used to elucidate our findings.

## 3. Results

The probe reaction chosen here for assessing the reactivity of Pt-doped Au surfaces was the dehydrogenation of CH_3_OH via O–H bond dissociation. Before focusing on this elementary reaction step, we first investigated the adsorption energies of all relevant chemical species on all Pt-free and Pt-enriched Au(111) surfaces, namely CH_3_OH, CH_3_O, OH, H, and O, as well as the coadsorption energies of CH_3_O/H, CH_3_O/OH, and CH_3_OH/O pairs.

**Figure 1:**
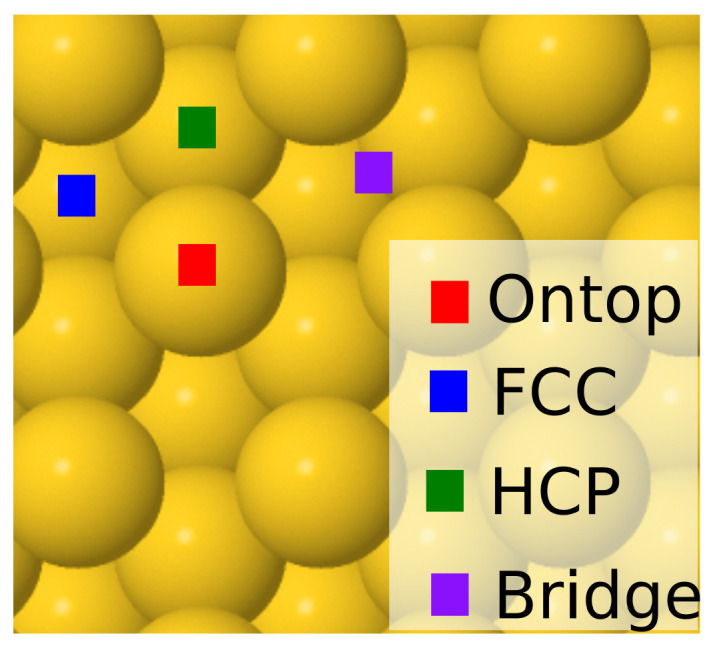
High-symmetry adsorption sites on Au(111) described in the text.

**Figure 2:**
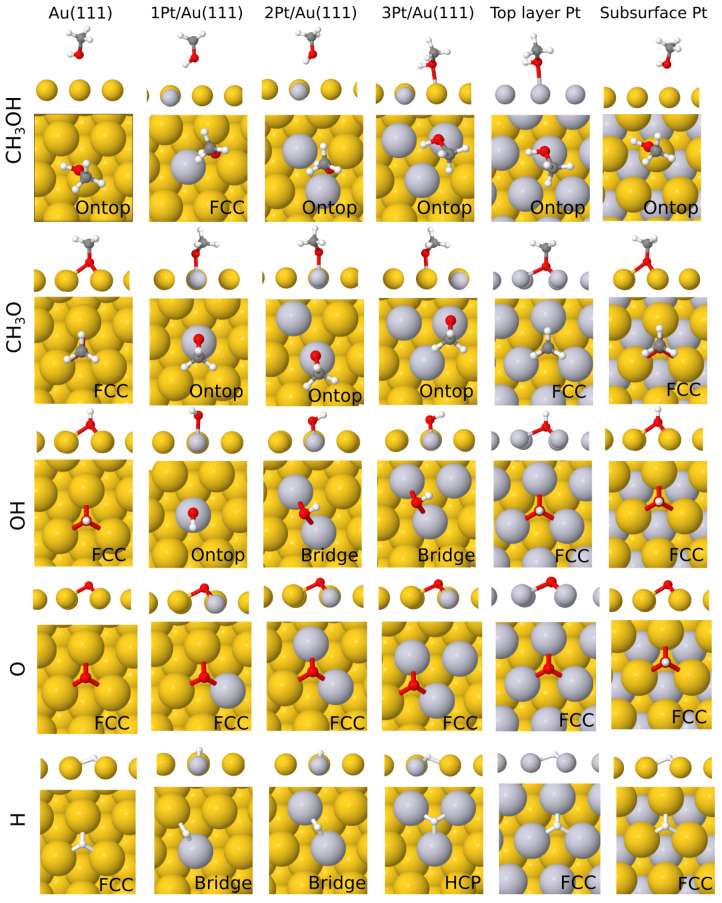
From top to bottom panels, the most stable adsorption configurations of CH_3_OH, CH_3_O, OH, O, and H for all surfaces investigated in this work. In the case of adsorption sites of similar stability (within the errors of the employed computational protocol), only one is arbitrarily reported.

In the case of single atomic or molecular adsorption, we considered four possible high-symmetry adsorption sites as our starting points for the geometry optimization: face-centered cubic (fcc), hexagonal closed packed (hcp), bridge (b), and on top (o) (see Figure 1). The fcc and hcp sites both refer to the centers of equilateral triangles on the Au(111) surface with the difference that the hcp site is located directly on top of a Au atom from the layer below, while the fcc hollow site is aligned with another hollow site on the layer below. The bridge and on-top sites, on the other hand, refer to positions halfway between and directly on top of surface atoms, respectively. Often, an adsorbate placed on one of these adsorption sites will migrate to another site during geometry optimization. Figure 2 depicts the most stable geometries of all adsorbates on the surfaces investigated, while the corresponding adsorption energies are reported in Table 1. The labels on each figure refer to adsorption sites at the end of the structural relaxation. For completeness, adsorption energies for all starting sites of the adsorbates are reported in full in Tables S1–S5 of the accompanying Supplementary Materials.

From an analysis of Table 1 (and Tables S1–S5 of the Supplementary Materials) we can observe that, with the exception of CH_3_OH, which was rather weakly adsorbed on all surfaces, all species showed strong affinity toward both the clean Au(111) and the various Pt-doped surfaces. For CH_3_OH, only the on-top adsorption position was moderately stabilized by Pt doping in the topmost layer (of about 0.3 eV), while on the clean Au(111) surface no adsorption site was strongly preferred over another. For CH_3_O adsorption, the two available high-symmetry sites (on top an fcc) were equally stable on clean Au(111). As the Pt:Au ratio increased, an enhancement in stability was observed, with the Pt overlayer displaying a clear preference towards fcc and hcp sites over the on-top site. Conversely, the presence of a Pt sublayer had little effect on methoxy adsorption compared to the clean Au surface. The adsorption energies of OH and the atomic species were already quite large on Au(111). However, a clear trend of increasing stability with an increasing surface Pt-doping concentration when going from 1 Pt/Au(111) to 3 Pt/Au(111) could easily be discerned, irrespective of the adsorption site. Pt doping in the form of a full Pt layer had the effect of either moderately decreasing the stability of the adsorbates (when in the form of an overlayer) or causing a noticeable decrease in the stability of the adsorbates (when in the form of a surface sublayer).

We examined and rationalized the trends observed in the adsorption energies (and later on the activation barriers) based on both the characteristics of the two individual metals and the synergistic effects that emerged from their interaction as components of the surface alloy. For CH_3_OH adsorption on 1 Pt/Au(111), 2 Pt/Au(111), and 3 Pt/Au(111) surfaces, the electronic effect due to alloying was particularly evident. Each substitutional Pt experienced a negative charge transfer of approximately −0.1 |e| from the Au atoms, as can be seen from the total Bader charges on Pt listed in Table S1. In each instance where an adsorbate formed a bond directly with one or more Pt atoms, there was an electron transfer from the Pt atoms in direct contact with the adsorbate to the adsorbate itself (Tables S1–S5). The accumulated negative charge was therefore passed over to the adsorbate. This accumulation of negative charge suggests that the positively charged hydrogen atom of the OH functional group of methanol experienced a stronger Coulomb attraction to the surface with an increasing Pt surface concentration. The Coulomb interaction resulting from this arrangement along with the strong charge transfer could explain both the increased adsorption energies and the destabilization of the O-H bond.

**Figure 3:**
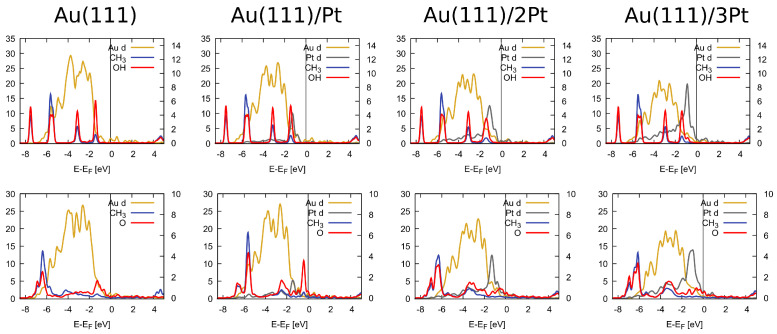
The partial density of states of CH_3_OH (**top** row) and CH_3_O (**bottom** row) adsorbed on Au(111), 1 Pt/Au, 2 Pt/Au, and 3 Pt/Au. Only the *d* states of the metals are shown, while all orbital contributions from the adsorbates are included. The vertical line indicates the position of the Fermi energy, which was set to be at zero. The left vertical axis refers to the metallic (Au and Pt) DOS, while the right vertical axis refers to the PDOS of the molecular fragments (CH_3_, OH, and O).

The electronic effect of Pt doping that enhanced the surface affinity towards methanol and methoxy could be observed in the PDOS profiles of adsorbed methanol and methoxy, reported in Figure 3. In contrast to the disjointed appearance of the Au *d* and methanol states on the bare Au(111) surface, the Pt-doped surfaces exhibited some overlap between the Pt *d* and methanol states just below the Fermi level. In the case of methoxy, on the other hand, the overlapping states in the energy range of 3–5 eV below the Fermi level were clearly visible.

A structural consequence of Pt doping is the strain effect on the Au lattice; this effect should be especially important in the case of a Pt overlayer. To isolate the effect of lattice strain from the others discussed above, we report in Table S6 of the Supplementary Materials the adsorption energies of methanol and methoxy on the Pt(111) surface constructed at both the Pt and Au lattice constants. While the adsorption energy of methanol remained largely unaffected (except for the on-top adsorption) by the added strain, the adsorption energy of methoxy was seen to increase by up to 0.5 eV for all adsorption sites, except for the on-top position, in the presence of lattice strain, which highlights the importance of strain in determining the stability of CH_3_O adsorption on the Pt overlayer. In the DFT study by Miao et al. [43], the reactivity of PdNi surface alloys towards ethanol dehydrogenation and oxidation was investigated in a similar manner to our work herein. A Ni layer was placed both on top of the Pd(111) surface and as the second layer from the top. In this case, the sublayer of Ni was found to reduce the reactivity, while the top layer was found to cause an increase. In this work as well, the changes in reactivity were rationalized by charge transfer and orbital overlap in the density of states.

According to the pioneering analysis method for metal alloys introduced by the Henkelman group [44], the adsorption behavior of molecules on the alloy surface can be explained via three effects: the ensemble effect (geometric), ligand effect (electronic), and strain effect. Our data could also be regrouped and analyzed in this manner. Table 1 shows that for the weak adsorption exhibited by CH_3_OH, the adsorption energy increased steadily as the local Pt concentration increased on the Au(111) surface. For all the other strongly bound species (CH_3_O, OH, O, and H), the alloys performed surprisingly better than the Pt overlayer. Such trends could be attributed to the geometry or ensemble effect. The electronic effect manifested itself in the d-band center, which, as seen in Figure 3, shifted towards the Fermi level as the surface Pt concentration increased. In addition, as listed in Table S1, each Pt atom acquired a negative charge from the surrounding Au atoms. Finally, the strain effect can be easily seen by considering our results for the Pt(111) surface calculated at the Au lattice constant.

Having developed an understanding of the alloying effects on the adsorption of single species, we next moved on to investigate whether the coadsorption of two species revealed any synergetic effects. The CH_3_OH/O, CH_3_O/OH, and CH_3_O/H coadsorption energies were calculated assuming that CH_3_OH and CH_3_O adsorbed at their most stable sites (as identified previously) and placing O, OH, and H adsorbates at the nearest four high-symmetry locations (hcp, fcc, bridge, and on top). The optimized geometries for the most stable coadsorption configurations considered are displayed in Figure S1, while the adsorption energies are listed in Table S7 of the Supplementary Materials. We also report the difference between the coadsorption energies calculated using Equation (4) and the sum of the individual most stable adsorption energies of the two species separately (each calculated using Equation (3)).

For all coadsorption pairs considered, a clear trend of increasing coadsorption energy was evident as a function of the increasing number of Pt atoms on the Au surface. For the CH_3_O/H pair, individual adsorption appeared to be slightly favored compared to coadsorption. For CH_3_O/OH, the presence of such an energy penalty depended on the surface. In contrast, for the CH_3_OH/O pair, there was a slight gain in energy upon coadsorption with the exception of the Pt overlayer.

**Figure 4:**
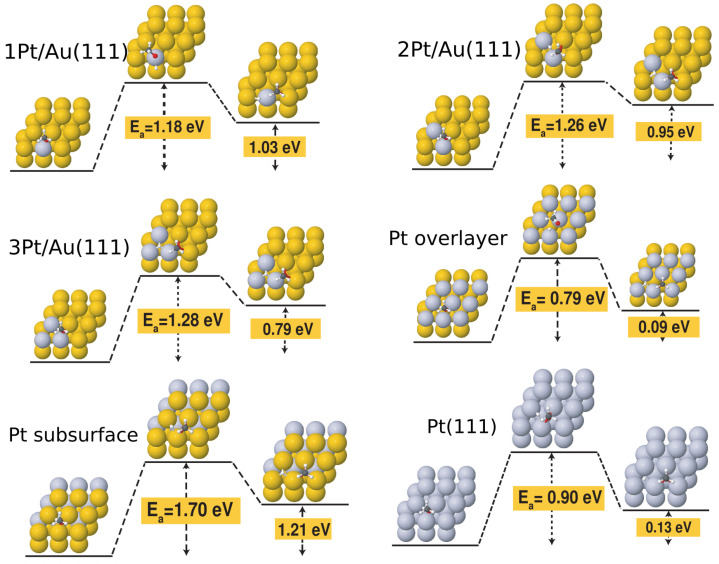
Initial, transition and final states of the methanol dehydrogenation reaction on Au(111), Pt/Au(111), 2 Pt/Au(111), 3 Pt/Au(111), 9 Pt/Au(111) (overlayer), subsurface Pt layer, and Pt(111).

Finally, we turn our attention to the effect of Pt doping on the activation barriers for the dehydrogenation reaction. Table 2 lists all the activation barriers and reaction energies calculated for the dissociation of the O-H bond in methanol, while Figure 4 illustrates the initial, transition, and final configurations of this elementary step on all Pt-doped surfaces investigated in this work. The PDOS profiles of the transition states are displayed in Figure S2, while the single imaginary frequencies of the transition states are listed in Table S8 of the Supplementary Materials. The benchmark activation barrier of 1.76 eV calculated for the Au(111) surface was in agreement with previous work [29]. With the introduction of the single substitutional Pt atom on the gold surface, we immediately observed a drop in the activation energy of about 0.6 eV. This was also consistent with the observation of a Pt *d* peak appearing just below the Fermi level, overlapping with a molecular state in Figure S2. The activation barrier was not significantly affected by switching from 1 Pt/Au to 2 Pt/Au (1.26 eV) and 3 Pt/Au (1.28 eV), but a significant drop was seen for the full Pt overlayer (0.79 eV). This was mirrored in the similarity of the PDOS profiles of the 2Pt/Au and 3Pt/Au shown in Figure S2. An interesting observation was that this activation energy value was lower than that calculated for the Pt(111) surface at the Au lattice constant (0.90 eV). This points towards a synergistic effect that can be seen in the PDOS of the transition state shown in Figure S3 of the Supplementary Materials, where the PDOS profile displays clear Pt/Au hybrid states near the Fermi energy.

Our activation barriers also compared favorably with previously reported values in the literature. The calculated barrier for H elimination from the OH group of methanol on the Pd/Au(111) alloy surface was reported to be 1.41 eV by Zhang et al. [7]. Our activation barrier for the same two-Pt configuration was 0.13 eV lower. The calculated ethanol dehydrogenation barriers on Rh/Au(111) and Pd/Au(111) surfaces showed a steady decrease of about 1 eV when increasing from one to nine dopant atoms on the surface [19]. While more modest, our activation barriers also showed a decrease with an increasing dopant concentration. Finally, Zhong et al. [21] reported a barrier for methanol dehydrogenation to methoxy of 0.20 eV on Ag-doped Au. In a DFT work conducted using a Pt single-atom catalyst on a Au(111) surface [45], the reaction barrier of the O-H bond scission reaction for ethanol was calculated as approximately 1.2 eV.

**Figure 5:**
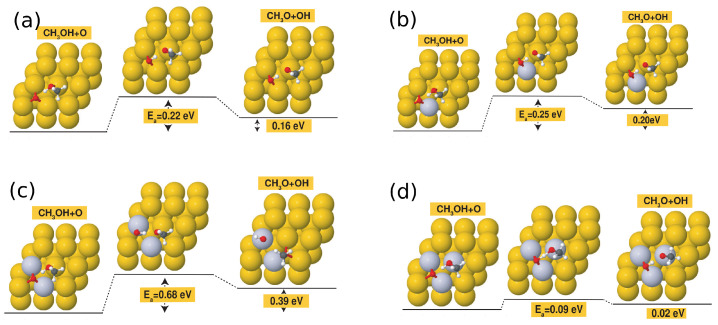
Initial, transition-state, and final configurations for the methanol dehydrogenation step on the following surfaces with preadsorbed atomic O: (**a**) bare Au(111) surface; (**b**) 1 Pt/Au surface; (**c**) 2 Pt/Au surface; and (**d**) 3 Pt/Au surface.

It is also important to note that Pt alloying can also be used to further reduce the activation barrier of methanol dehydrogenation on Au surfaces with preadsorbed atomic oxygen, as can be seen from Figure 5. In particular, the activation barrier was reduced to as low as 0.09 eV in the case of the 3Pt/Au surface with preadsorbed atomic oxygen.

Overall, our results were in agreement with the literature. For instance, Xu et al. [29] found that the adsorption energy of methanol on a pristine Au(111) surface was 0.15 eV, while in the presence of an oxygen atom, the adsorption energy was 0.44 eV. Furthermore, the O–H bond dissociation activation energies also agreed closely. In Xu’s work, the activation barriers in the absence and presence of preadsorbed O were calculated to be 1.58 eV and 0.41 eV, respectively. Finally, the thermodynamic reaction energy in the presence and absence of surface O was calculated to be 1.33 eV and 0.27 eV, respectively, with both cases being endothermic. Once again, these values were very close to our calculated thermodynamics energies of 1.34 eV and 0.16 eV.

## 4. Conclusions

In this work, using first-principles density functional theory, we investigated the effect of Pt surface doping on the reactivity of a Au(111) surface towards methanol dehydrogenation. The effect of increasing the Pt-doping concentration of the surface on the activation barrier of this elementary step was systematically studied by substituting one, two, and three surface Au atoms with Pt. In addition, Pt doping in the form of a full Pt overlayer and a full Pt sublayer was also investigated. The calculation of the adsorption energies of CH_3_OH, CH_3_O, OH, O, and H on bare Au(111) and doped surfaces indicated that the affinity of the metallic surface towards these adsorbates increased with an increasing Pt concentration on the surface. This effect of Pt doping was attributed to a charge accumulation phenomenon that steadily built up on the surface Pt atoms. With an increasing Pt concentration, the *d*-center of the surface was seen to be displaced towards the Fermi level. The effect of the strain induced by the doping was also investigated, and it was seen that lattice strain stabilized the adsorbed CH_3_O species (by about 0.5 eV on a full Pt overlayer).

A key result of this work was the finding that the activation barrier for the dehydrogenation reaction was already reduced, by more than 30% compared to bare Au(111), for a small surface doping concentration of Pt. When the elementary step was studied on a full Pt overlayer, the activation barrier was further reduced to about 0.80 eV, which was lower than the activation barrier calculated on bare Pt(111). Therefore, even at the limit of full Pt coverage, the Au and Pt components acted in concert to create a more reactive catalyst. This study also pointed out the important role of preadsorbed atomic oxygen on further lowering the reaction barrier of the dehydrogenation step: when oxygen pretreatment was coupled with Pt doping, the activation barrier of the elementary step reached a value as low as 0.10 eV.

## Figures and Tables

**Table 1:** Adsorption energies (in eV) corresponding to the most stable adsorption site (reported in parenthesis) for CH_3_OH, CH_3_O, OH, O, and H adsorbates on bare Au(111) surface and on all Pt-doped surfaces investigated in this work.

	Au(111)	1 Pt/Au	2 Pt/Au	3 Pt/Au	Pt Overlayer	Pt Sublayer
CH_3_OH	−0.11 (o)	−0.12 (fcc)	−0.13 (o)	−0.27 (o)	−0.38 (o)	−0.12 (o)
CH_3_O	−1.75 (fcc)	−1.88 (o)	−1.87 (o)	−1.92 (o)	−2.24 (fcc)	−1.77 (fcc)
OH	−2.66 (fcc)	−2.73 (o)	−2.80 (b)	−2.82 (b)	−2.65 (fcc)	−2.19 (fcc)
O	−5.07 (fcc)	−5.24 (fcc)	−5.43 (fcc)	−5.40 (fcc)	−4.67 (fcc)	−3.46 (fcc)
H	−3.18 (fcc)	−3.46 (b)	−3.74 (b)	−3.84 (hcp)	−3.04 (fcc)	−2.26 (fcc)

**Table 2:** Activation barriers (E${}_{a}$, in eV) and reaction energies (E${}_{r}$, in eV) for the methanol dehydrogenation step on the surfaces investigated in this work.

Surface	E${}_{a}$	E${}_{r}$
Au(111)	1.76	1.34
1 Pt/Au	1.18	1.03
2 Pt/Au	1.26	0.95
3 Pt/Au	1.28	0.79
Pt overlayer	0.79	0.08
Pt sublayer	1.70	1.21
Pt(111) ${}^{a}$	0.90	0.13

${}^{a}$ At the Au lattice constant.

## Data Availability

The data that support the findings of this study are available from the corresponding author upon reasonable request.

## Supplementary Materials

The following supporting information can be downloaded at: https://www.mdpi.com/article/10.3390/molecules28237928/s1, Figure S1: The most stable coadsorption geometries of CH_3_O/H (top panels), CH_3_O/OH (middle panels) and CH_3_OH/O (bottom panels) on different Au(111) surfaces, namely, Au(111), 1Pt/Au, 2Pt/Au, 3Pt/Au and with a single Pt overlayer; Figure S2: PDOS profiles of the transition state of the dehydrogenation reaction on Au(111) (a), Au(111)/Pt (b), Au(111)/2Pt (c), Au(111)/3Pt, (d) Au(111)/9Pt (overlayer), and (e) subsurface Pt layer; Figure S3: PDOS profiles corresponding to the initial, transition state and final configurations for the methanol dehydrogenation step on the Au(111) supported Pt overlayer. Au states refer to the Au atoms on the layer below the Pt overlayer; Table S1: Adsorption energies (in eV) of CH_3_OH on bare Au(111) and on all Pt-doped surfaces investigated in this work (o≡ontop, b≡bridge). The total Bader partial charge on the Pt atoms (in units of the elementary charge, |e|) is also reported in parenthesis; Table S2: Adsorption energies (in eV) of CH_3_O on bare Au(111) and on all Pt-doped surfaces investigated in this work (o≡ontop, b≡bridge). The total Bader partial charge on the Pt atoms (in units of the elementary charge, |e|) is also reported in parenthesis; Table S3: Adsorption energies (in eV) of OH on bare Au(111) and on all Pt-doped surfaces investigated in this work (o≡ontop, b≡bridge). The total Bader partial charge on the Pt atoms (in units of the elementary charge, |e|) is also reported in parenthesis; Table S4: Adsorption energies (in eV) of atomic O on bare Au(111) and on all Pt-doped surfaces investigated in this work. The total Bader partial charge on the Pt atoms (in units of the elementary charge, |e|) is also reported in parenthesis; Table S5: Adsorption energies (in eV) of atomic H on bare Au(111) and on all Pt-doped surfaces investigated in this work (o≡ontop, b≡bridge). The total Bader partial charge on the Pt atoms (in units of the elementary charge, |e|) is also reported in parenthesis; Table S6: Adsorption energies (in eV) of CH_3_OH and CH_3_O on the Pt(111) surface constructed at the bulk Pt lattice constant. Values in parenthesis refer to the same surface constructed at the bulk lattice constant of Au; Table S7: The most stable coadsorption energies (in eV) of CH_3_O and CH_3_OH with H, OH, and O on bare Au(111) and Pt-doped surfaces considered in this work. The differences with respect to the sum of the most stable adsorption energies of the two co-adsorbates are given in parantheses. A negative value signifies a more stable coadsorption scenario with respect to individual adsorption. The adsorption geometries corresponding to the data in this table are given in Figure S1; Table S8: Absolute values of the imaginary frequencies (in cm${}^{-1}$) of the transition states of the methanol dehydrogenation reactions on Au(111), and Pt-decorated Au(111) surfaces.

## Abbreviations

The following abbreviations are used in this manuscript:

DFT	Density functional theory
GGA	Generalized gradient approximation
PW-91	Perdew–Wang exchange correlation potential
BFGS	Broyden–Fletcher–Goldfarb–Shanno
CI-NEB	Climbing image nudged elastic band method
PDOS	Partial density of states
