# Supplementary material for: Effect of Surface Pt Doping on the Reactivity of Au(111) Surfaces towards Methanol Dehydrogenation: A First-Principles Density Functional Theory Investigation
Source: Molecules. 2023 Dec 4;28(23):7928. doi: 10.3390/molecules28237928 (PMC10707965; doi:10.3390/molecules28237928)
Supplement: Supplementary file 1 [file molecules-28-07928-s001.zip › molecules-2702753-supplementary.pdf]

Supplementary Material for: Effect of surface Pt doping on the reactivity of the Au(111) surface towards methanol dehydrogenation: a first-principles Density Functional Theory investigation

Merve Demirtas,[†,¶] Hande Ustunel,[*,†] and Daniele Toffoli[*,‡,§]

†*Department of Physics, Middle East Technical University, Dumlupinar Blv 1, 06800, Ankara, Turkey*

‡*Dipartimento di Scienze Chimiche e Farmaceutiche, Università degli Studi di Trieste,Via L. Giorgieri 1, I-34127, Trieste, Italy*

¶*Network Technologies Department, TUBITAK ULAKBIM, 06800, Ankara, Turkey*

§*IOM-CNR, Istituto Officina dei Materiali-CNR, S.S.14, Km 163.5, 34149 Trieste, Italy*

E-mail: ustunel@metu.edu.tr; toffoli@units.it

The Tables in this section collect, for each adsorbate, the adsorption energies for the four high-symmetry adsorption sites considered in this study for both bare Au(111) and Pt doped surfaces. Missing entries in the Tables indicate that, none of the starting geometries were found to yield a final geometry anchored at the symmetry point in question.

Table S1: Adsorption energies (in eV) of CH_3OH on bare Au(111) and on all Pt-doped surfaces investigated in this work (o≡ontop, b≡bridge). The total Bader partial charge on the Pt atoms (in units of the elementary charge, $|e|$) is also reported in parenthesis.

site	Au(111)	1Pt/Au	2Pt/Au	3Pt/Au	Pt overlayer	Pt sublayer
o	-0.11	-0.12(-0.12)	-0.13(-0.25)	-0.27(-0.27)	-0.38(-0.62)	-0.12(-0.15)
fcc	-0.09	-0.12(-0.14)	-0.13(-0.25)	-0.13(-0.34)	-0.13(-0.62)	-0.10(-0.14)
hcp	-0.10	-0.11(-0.10)	-0.13(-0.24)	—	-0.13(-0.56)	—
b	-0.11	-0.10(-0.08)	-0.13(-0.22)	-0.13(-0.27)	-0.15(-0.62)	-0.12(-0.15)

Table S2: Adsorption energies (in eV) of CH_3O on bare Au(111) and on all Pt-doped surfaces investigated in this work (o≡ontop, b≡bridge). The total Bader partial charge on the Pt atoms (in units of the elementary charge, $|e|$) is also reported in parenthesis.

site	Au(111)	1Pt/Au	2Pt/Au	3Pt/Au	Pt overlayer	Pt sublayer
o	-1.74	-1.88(-0.10)	-1.87(+0.17)	-1.92(+0.09)	-1.95(-0.19)	-1.54(-0.19)
fcc	-1.75	-1.73(+0.10)	-1.70(+0.02)	—	-2.24(-0.11)	-1.77(-0.17)
hcp	—	-1.88(+0.23)	—	—	-2.23(-0.16)	-1.64(-0.15)
b	—	-1.74(+0.22)	-1.86(+0.17)	-1.91(+0.17)	—	-1.66(-0.18)

Table S3: Adsorption energies (in eV) of OH on bare Au(111) and on all Pt-doped surfaces investigated in this work (o≡ontop, b≡bridge). The total Bader partial charge on the Pt atoms (in units of the elementary charge, $|e|$) is also reported in parenthesis.

site	Au(111)	1Pt/Au	2Pt/Au	3Pt/Au	Pt overlayer	Pt sublayer
o	-2.41	-2.73(+0.22)	-2.27(-0.19)	-2.76(+0.06)	-2.93(-0.15)	-2.63(-0.18)
fcc	-2.66	-2.61(+0.08)	—	–	-3.13(+0.00)	-2.67(-0.19)
hcp	-2.53	—	—	—	-2.88(-0.11)	-2.44(-0.16)
b	—	—	-2.80(+0.20)	-2.82(+0.09)	-2.76(-0.08)	-2.46(-0.19)

Table S4: Adsorption energies (in eV) of atomic O on bare Au(111) and on all Pt-doped surfaces investigated in this work. The total Bader partial charge on the Pt atoms (in units of the elementary charge, $|e|$) is also reported in parenthesis.

site	Au(111)	1Pt/Au	2Pt/Au	3Pt/Au	Pt overlayer	Pt sublayer
o	—	—	—	—	—	—
fcc	-5.07	-5.24(+0.19)	-5.43(+0.36)	-5.40(+0.29)	-6.28(+0.23)	-5.07(-0.19)
hcp	-5.05	-5.22(+0.20)	-5.17(+0.36)	-5.36(+0.47)	-6.06(+0.19)	-4.72(-0.20)
b	—	—	—	—	—	—

Table S5: Adsorption energies (in eV) of atomic H on bare Au(111) and on all Pt-doped surfaces investigated in this work (o≡ontop, b≡bridge). The total Bader partial charge on the Pt atoms (in units of the elementary charge, $|e|$) is also reported in parenthesis.

site	Au(111)	1Pt/Au	2Pt/Au	3Pt/Au	Pt overlayer	Pt sublayer
o	-3.01	-2.98(-0.10)	-2.99(-0.20)	-3.65(-0.20)	-3.72(-0.50)	-3.21(-0.24)
fcc	-3.18	-3.45(-0.05)	—	—	-4.11(-0.48)	-3.33(-0.24)
hcp	-3.15	-3.45(-0.06)	—	-3.84(-0.18)	-4.06(-0.51)	—
b	-3.13	-3.46(-0.05)	-3.74(-0.10)	—	—	—

Table S6: Adsorption energies (in eV) of CH_3OH and CH_3O on the Pt(111) surface constructed at the bulk Pt lattice constant. Values in parenthesis refer to the same surface constructed at the bulk lattice constant of Au.

site	CH_3OH	CH_3O
hcp	-0.12(-0.15)	-1.92(-2.43)
fcc	-0.12(-0.13)	-1.82(-2.34)
b	-0.12(-0.17)	-1.92(-2.43)
o	-0.17(-0.40)	-1.99(-2.16)

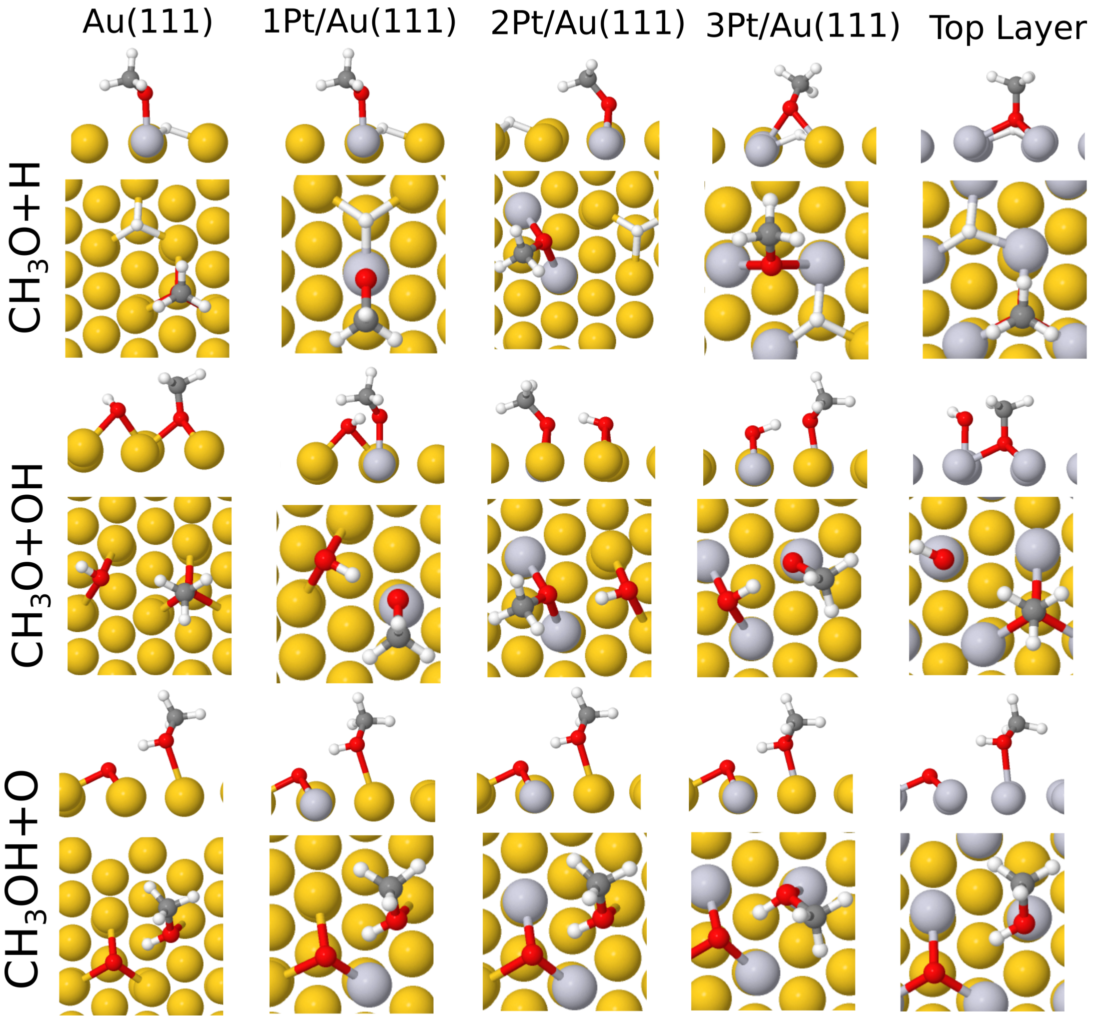

Figure S1: The most stable coadsorption geometries of CH_3O/H (top panels), CH_3O/OH (middle panels) and CH_3OH/O (bottom panels) on different Au(111) surfaces, namely, Au(111), 1Pt/Au, 2Pt/Au, 3Pt/Au and with a single Pt overlayer.

Table S7: The most stable coadsorption energies (in eV) of CH_3O and CH_3OH with H, OH, and O on bare Au(111) and Pt-doped surfaces considered in this work. The differences with respect to the sum of the most stable adsorption energies of the two co-adsorbates are given in parantheses. A negative value signifies a more stable coadsorption scenario with respect to individual adsorption. The adsorption geometries corresponding to the data in this table are given in Figure S1.

coads. pair	Au(111)	1Pt/Au	2Pt/Au	3Pt/Au	Pt overlayer
CH_3O+H	-4.89 (0.02)	-5.09 (0.25)	-5.39 (0.22)	-5.38 (0.38)	-6.17 (0.18)
CH_3O+OH	-4.38 (0.03)	-4.68 (-0.07)	-4.45 (0.22)	-5.03 (-0.28)	-5.08 (0.29)
CH_3OH+O	-5.46 (-0.27)	-5.61 (-0.27)	-5.80 (-0.24)	-5.87 (-0.20)	-6.64 (0.02)

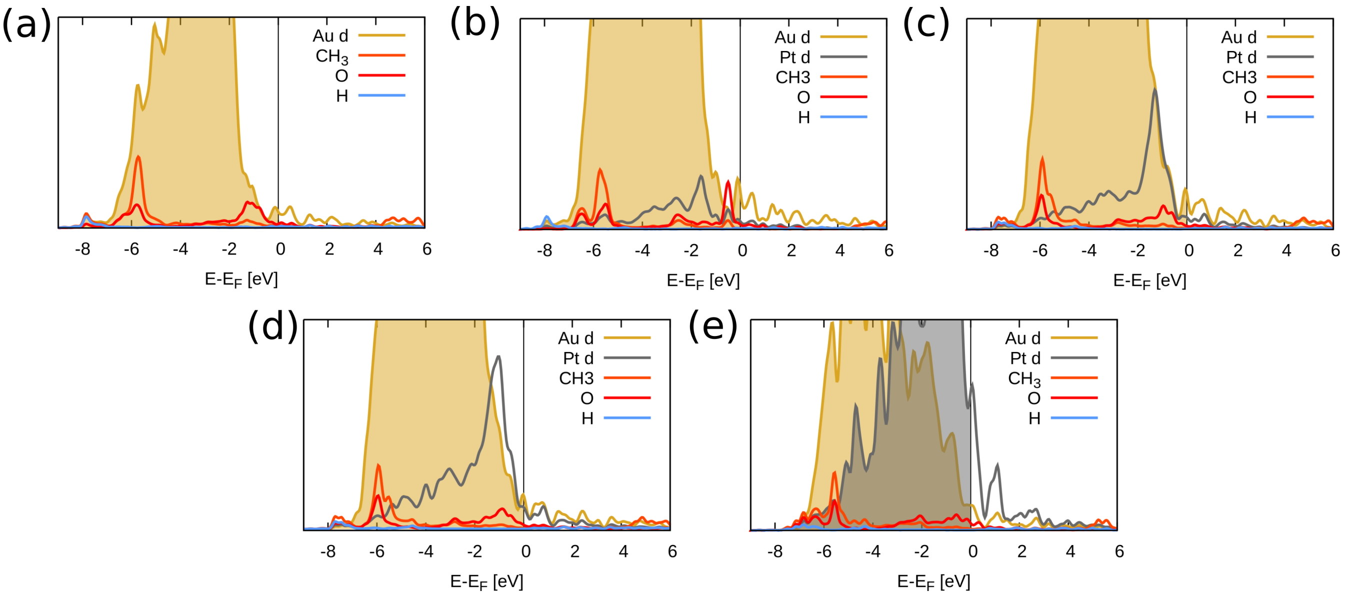

Figure S2: PDOS profiles of the transition state of the dehydrogenation reaction on Au(111) (a), Au(111)/Pt (b), Au(111)/2Pt (c), Au(111)/3Pt, (d) Au(111)/9Pt (overlayer), and (e) subsurface Pt layer.

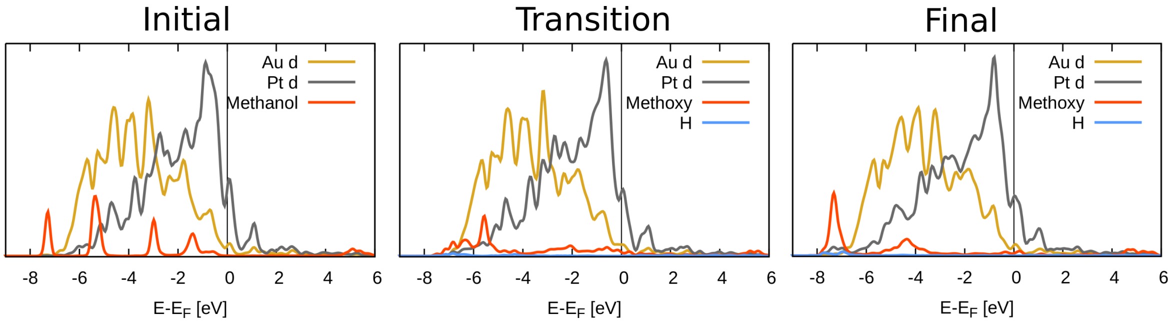

Figure S3: PDOS profiles corresponding to the initial, transition state and final configurations for the methanol dehydrogenation step on the Au(111) supported Pt overlayer. Au states refer to the Au atoms on the layer below the Pt overlayer.

Table S8: Absolute values of the imaginary frequencies (in cm^{-1}) of the transition states of the methanol dehydrogenation reactions on Au(111), and Pt-decorated Au(111) surfaces

Surface	$\sqrt{\|\omega^2\|}$ [cm^{-1}]
Au(111)	314.4
Au(111)/Pt	155.7
Au(111)/2Pt	280.5
Au(111)/3Pt	348.2
Pt overlayer	616.7
Pt sublayer	489.7
